# Bladder Endometriosis as Part of Complex Pelvic Deep Endometriosis: Surgical Challenges and Outcomes in a Reference Center

**DOI:** 10.3390/jcm15051995

**Published:** 2026-03-05

**Authors:** Maja Mrugała, Marek Fiutowski, Alicja Dąbrowska, Krzysztof Nowak, Ewa Milnerowicz-Nabzdyk

**Affiliations:** Oncological Gynecology Department and Reference Endometriosis Center, Center of Oncology in Opole, Opole University, 45-061 Opole, Poland; maja.mrugala@gmail.com (M.M.); knowakmd@gmail.com (K.N.)

**Keywords:** bladder endometriosis, deep endometriosis, urinary tract endometriosis, laparoscopy, bladder resection

## Abstract

**Objective**: To analyze multiple aspects of advanced bladder endometriosis surgery, based on the experience of an endometriosis reference center. **Methods**: This retrospective/prospective study included 80 consecutive patients with deep bladder endometriosis treated with laparoscopic surgery. **Results**: In 96.3% of cases, bladder endometriosis coexisted with other organ involvement: bowel (87.5%), uterus (61.3%), and ureters (37.5%); isolated bladder lesions occurred in 3.7%. Full-thickness bladder infiltration occurred in 36.4% of patients, and 71.8% had a history of surgery. The most frequent preoperative symptoms related to multiorgan involvement were dysmenorrhea (88.7%), dyschezia (75.0%), and dyspareunia (55.7%). Dysuria (55.7%), pollakiuria (17.9%), and urinary urgency (9.0%) were also reported. Shaving was performed in 45.0% of cases, resection in 40.0%, skinning in 15.0%, with two rare cases requiring bladder augmentation with bowel insert. Of all multiorgan surgeries (96.3% of cases), the most complex 30% were performed by a bi-disciplinary team of gynecologists and urologists. Postoperative complications occurred in 8 patients (10%) and were significantly associated with larger lesions, full-thickness infiltration, trigonum involvement, multiple organs opened, and prior surgery. **Conclusions**: Laparoscopic management of bladder endometriosis is feasible and effective, even in complex cases. Postoperative complications were linked to disease complexity but remained low, likely due to protective techniques used by the reference team. Optimal outcomes for the most difficult cases are more likely when procedures are performed by a bi-disciplinary team involving both oncological gynecologists specialized in deep endometriosis surgery and urologists. Given the heterogeneous clinical profiles of bladder endometriosis, treatment should be carried out in specialized centers where individualized surgical strategies can be implemented.

## 1. Introduction

Endometriosis is a chronic, estrogen-dependent inflammatory disease characterized by the presence of endometrial-like tissue outside the uterus, affecting approximately 10% of women of reproductive age [1,2]. Deep endometriosis (DE), defined as infiltration penetrating more than 5 mm under the peritoneal surface, represents the most aggressive form of the disease [3]. Urinary tract endometriosis is diagnosed in about 1–12% of women with endometriosis, with the bladder being the most frequently affected organ (approximately 85% of urinary tract cases) [4,5].

Bladder endometriosis is associated with significant morbidity. The clinical presentation typically includes lower urinary tract symptoms (LUTS) such as frequency, urgency, dysuria, and occasionally hematuria [6]. Characteristically, these symptoms are often constant but significantly exacerbated during menstruation (catamenial symptoms). However, bladder nodules are rarely isolated and typically coexist with other deep lesions in the pelvis, such as colorectal or ureteral endometriosis. Consequently, specific bladder complaints are frequently overshadowed by severe dysmenorrhea, chronic pelvic pain, and dyspareunia resulting from the multiorgan nature of the disease [7]. This complex symptomatology often leads to diagnostic delays and multiple ineffective surgical interventions before the correct diagnosis is made.

Surgical management of bladder DE is challenging and requires a balance between radical removal of the lesion to prevent recurrence and organ preservation to maintain urinary function. It is often part of a complex, multidisciplinary procedure involving bowel or ureteral resection [8,9].

The primary aim of this study was to evaluate the clinical and anatomical characteristics of bladder DE and to analyze the surgical management strategies in a large cohort of patients. The secondary endpoints included the assessment of coexisting multiorgan DE lesions and the evaluation of perioperative outcomes (including complication rates and hemoglobin drop), positioning bladder surgery within the context of radical multidisciplinary treatment.

## 2. Materials and Methods

This was a retrospective/prospective cohort study. The analysis included 80 consecutive patients with bladder endometriosis who underwent surgery at the Opole Oncology Center (OCO), Opole, Poland, between 13 May 2020, and 19 May 2025. This center serves as both a reference center for gynecological oncology treatment and a referral center for patients with advanced DE. The study protocol was approved by the Ethics Committee at the University of Opole (approval no: UO/0001/KB/2023). These cases were selected from a total of all 125 cases of DE involving the urinary tract. The relatively large number of patients treated for this highly specialized condition is due to the lack of designated referral centers for this disease in Poland as of 30 June 2025. During these five years, OCO performed one of the highest, if not the highest, numbers of surgeries in Poland for complex DE with urinary tract involvement. The endometriosis team consisted of the first surgeon, an oncological gynecologist specialized in DE, with a volume of complex DE cases of around 700; the team of oncological gynecologists trained in DE, with a volume of around 250 cases each; and a dedicated urologist, who was invited to participate in elective surgeries.

The retrieved data were exported to a Microsoft Excel spreadsheet (Microsoft Excel 2013; Microsoft Corp., Redmond, WA, USA) for further processing. Statistical analyses were performed using MedCalc software, version 19.5.3 (MedCalc Software Ltd., Ostend, Belgium). Data were presented as means with standard deviations (SD) or as counts with corresponding percentages. The distribution of continuous variables was assessed using the Shapiro–Wilk test. Group comparisons were performed using the Mann–Whitney U test or the Kruskal–Wallis test, depending on the number of groups. Categorical variables were analyzed using the Chi-square test. A *p*-value of <0.05 was considered statistically significant.

## 3. Results

### 3.1. Patient Characteristics

Of the 125 patients, 80 had bladder endometriosis and were included in the analysis. The average age of the included patients was 36.85 ± 6.18 years. The average BMI was 23.10 ± 3.33 kg/m^2^ (for one patient, BMI data were missing), which falls within the normal range. Only two patients were classified as obese, with a BMI of over 30. The detailed patient characteristics, including the hospitalization duration, are presented in Table 1.

**Figure 1:**
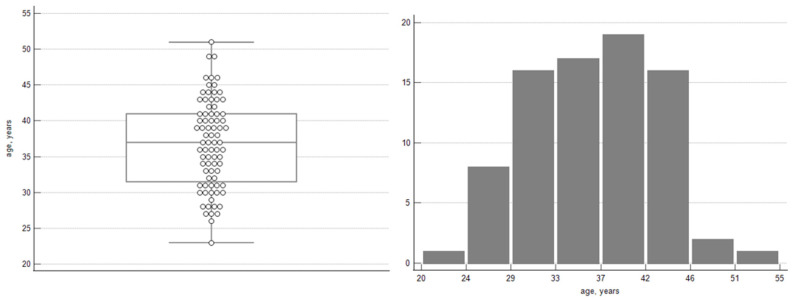
Age distribution presented using a box-and-whisker plot and a histogram.

As shown in Figure 1, the average age of women who underwent surgery corresponds to full adulthood, with the majority being middle-aged. The time from the diagnosis done by the first surgeon who performed consultation in outpatient clinics to surgery ranged from 1 to 4 months on average, depending on the urgency of the surgery.

**Figure 2:**
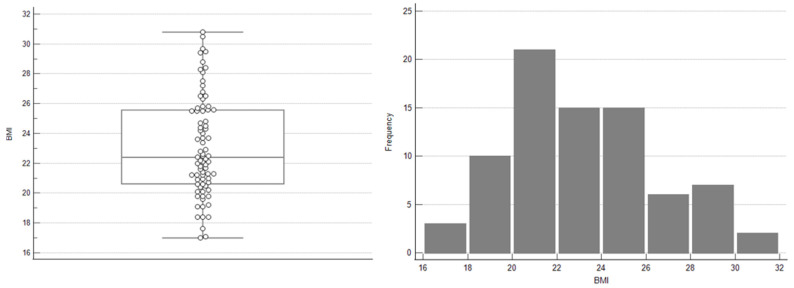
BMI distribution presented using a box-and-whisker plot and a histogram.

The majority of patients (over 63%) had a normal body weight, while nearly 27% were classified as overweight. Obesity was observed in only 2.5% of cases. Compared to data from the general population of reproductive-aged women in Poland, these findings suggest that women with endometriotic bladder lesions tend to have lower body weight than their age-matched peers in the general population. The distribution of BMI values is shown in Figure 2.

### 3.2. Eligibility Criteria for Urinary Tract Surgery

The following inclusion criteria were applied, resulting in the enrollment of the following patients:

- Symptomatic patients with a bladder lesion identified on ultrasound or MRI, subsequently confirmed as endometriosis by postoperative histopathological examination.
- Patients scheduled for surgery due to DE involving symptomatic full-thickness intestinal lesions, in whom bladder infiltration was discovered intraoperatively and confirmed as endometriosis by histopathological examination.
- Patients undergoing follow-up for DE, in whom hydronephrosis or dilation of the pelvicalyceal system was found as a secondary sign of endometriotic infiltration, with intraoperative confirmation of bladder or ureteral involvement.
- Patients in whom a bladder lesion was detected incidentally during abdominal surgery for endometriosis of other organs, subsequently excised and confirmed as endometriosis by histopathological examination.

The following patients were excluded:

- Patients with DE in whom no bladder involvement was identified either preoperatively or intraoperatively.
- Patients in whom histopathological examination did not confirm DE affecting the urinary tract.

### 3.3. Examinations Used to Verify Eligibility Criteria

Preoperative evaluation of patients qualified for surgery was performed to establish the diagnosis and assess disease severity using the following examinations:

1. Transvaginal ultrasound (TVUS)—mandatory;
2. Renal ultrasound—mandatory;
3. Pelvic MRI—mandatory;
4. CT urography (UroCT)—in selected cases, when clinically indicated;
5. Renal scintigraphy—in selected cases, when clinically indicated.

The first two tests (TVUS and renal ultrasound) were performed routinely in all patients with DE. Pelvic MRI was additionally performed in all patients qualified for surgery. UroCT was used when urinary tract involvement was suspected, and the first three imaging modalities were insufficient. Renal scintigraphy was performed in cases of suspected severe renal dysfunction or necrosis, most commonly in patients with massive hydronephrosis or sonographic features suggestive of an atrophic kidney.

### 3.4. Overview of Bladder Surgery Types

1. Bladder shaving surgery is a procedure involving excision of endometriosis tissue from the bladder wall, performed in cases of partial-thickness lesions that do not infiltrate the bladder muscle or involve it only minimally.
2. Mucosal skinning surgery is a procedure involving the removal of an endometriosis lesion affecting the deeper layers of the bladder wall, including the muscle layer, possibly up to the full thickness, but without infiltration of the bladder wall mucosa. These procedures are performed without opening the bladder lumen.
3. Resection of bladder endometriosis is a procedure involving the resection of a full-thickness lesion of the bladder wall. In such cases, opening the bladder lumen is required, as the lesion infiltrates all layers, including the mucosa. These procedures constitute full-thickness resections of the bladder wall.
4. Surgery involving bladder augmentation with an intestinal segment is a procedure in which a full-thickness lesion of the bladder wall requires augmentation of the bladder volume with an intestinal patch. We mention this surgical option because one patient, due to extensive involvement of both bladder walls, required such an advanced procedure using a segment of the small intestine.

Most complex elective surgeries (n = 24; 30%) were performed with the assistance of a urologist. Similarly, repair elective procedures were carried out either with an assisting urologist or by a urologist with the support of a gynecologist. However, a considerable number of surgeries were performed solely by a team of gynecologic oncologists specializing in DE (n = 56; 70%), without urologist involvement.

Surgical treatment of bladder endometriosis is typically of shorter duration; however, in this study, all patients had multiorgan involvement, which resulted in prolonged operative times, with a mean duration of approximately 3 h and 20 min. However, it should be noted that the vast majority of patients also presented with additional infiltrations involving the large intestine, uterus, parametrium, and ureters. As a result, there were complex, multiorgan procedures, which were discussed later. Another factor contributing to the extended operative time was the history of multiple previous surgeries, which increased the technical difficulty of the procedure; this aspect is also analyzed separately.

### 3.5. Previous Surgeries

Considering the hypothesis that bladder lesions occur more frequently in women with a history of prior surgical interventions, the number of previous surgical procedures was analyzed in the study group.

The majority of patients had a history of prior surgical interventions involving the adnexa, uterus, and abdominal cavity, which added to the complexity of the procedures performed. We investigated the relationship between the number of uterine surgeries, specifically cesarean sections, and the presence of bladder infiltration, to compare our findings with the existing literature. Table 2 presents the characteristics of the operated patients based on the type of previous interventions: all surgeries involving the pelvis and abdominal cavity, uterine procedures excluding cesarean sections, cesarean sections, and the total number of uterine incisions and procedures.

Among the 78 patients (2 cases had missing data) included in the study, 71.8% had undergone at least one prior surgical procedure, and 71.4% had a history of surgery involving the intestines, ureters, or reproductive organs. Surgeries specifically involving the bladder or ureters were reported in 11.4% of patients. Additionally, 33.8% of patients had previously undergone uterine surgery, and 27.8% had delivered babies via Cesarean section.

### 3.6. Endometriosis Tumors of the Bladder

The size of the lesions ranged from 0.5 to 9 cm, with a mean of 2.07 ± 1.24 cm. The difference in lesion size across four lesion location groups (anterior wall, posterior wall, fundus, and trigonum) was not statistically significant (*p* = 0.0850). The most common location of endometriosis lesions was the bladder wall (anterior or posterior), observed in 57 out of 80 cases (71.3%), followed by the fundus (n = 14; 17.5%) and the trigonum (n = 8; 10.0%).

Partial infiltration of the bladder wall was observed in 50 cases (62.5%), while full-thickness infiltration was present in 30 cases (37.5%). The difference in distribution of infiltration depth across lesion location groups was not statistically significant (*p* = 0.3347). In general, partially infiltrating lesions were managed with shaving or skinning techniques, whereas lesions with full-thickness bladder wall infiltration required surgical resection.

Among the surgical techniques used, shaving was the most frequently performed procedure, accounting for 45.0% of cases (n = 36). This was followed by resection in 40.0% (n = 32) and skinning in 15.0% (n = 12). The difference in distribution of the type of surgery across lesion location groups was not statistically significant (*p* = 0.7533). The detailed characteristics of endometriosis tumors of the bladder are presented in Table 3.

In 77 out of 80 cases (96.3%), bladder endometriosis was associated with endometriosis in other locations. The most common coexisting sites were the intestines (70/80; 87.5%), uterus (49/80; 61.3%), and ureters (30/80; 37.5%). Among these 77 cases with multi-site involvement, 28 (36.4%) presented with full-thickness bladder wall infiltration.

Surgery for endometriosis involving both the bladder and ureter was performed in 30 patients, with a urologist present in 16 of these cases (53.3%).

### 3.7. Features of Surgery—Duration, Hemoglobin Drop, Lesion Size

Differences in hemoglobin drop and duration of surgery between groups categorized by lesion location, type of infiltration, type of surgery, and history of previous surgery were not statistically significant. Correlation between hemoglobin drop and lesion size (rho = −0.0285; *p* = 0.8067). However, a significant positive correlation was observed between surgery duration and lesion size (rho = 0.332; *p* = 0.0028), but not significant between hemoglobin drop and lesion size (rho = −0.0285; *p* = 0.8067). Lesions involving the full thickness of the bladder wall were significantly larger than those limited to partial wall infiltration (2.92 ± 1.41 cm vs. 1.56 ± 0.76 cm; *p* < 0.0001). Additionally, smaller lesions were treated using the least invasive procedures, while larger lesions required more extensive surgical approaches.

In our analysis, the most complex procedures, involving advanced urological interventions for ureter involvement alongside bowel surgery, were managed by a bi-disciplinary gynecology–urology team. They involved lesions that were numerically larger (2.81 ± 1.61 cm vs. 1.75 ± 0.88 cm), which contributed to significantly longer operative times (341.65 ± 145.82 min vs. 79.82 ± 101.41 min; *p* = 0.0013) and slightly greater hemoglobin drop (2.60 ± 1.17 vs. 2.52 ± 1.08). These findings are detailed in Table 4.

### 3.8. Coexistence of Bladder Endometriosis with Endometriosis in Other Organ Locations

Bladder endometriosis surgeries are typically complex, involving not only the urinary tract but also two or more additional organs. In the present study, isolated bladder endometriosis was observed in only 3 out of 80 cases (3.8%). In the remaining cases, bladder involvement coexisted with lesions in other organs, most commonly the intestines (70/80; 87.5%), uterus (49/80; 61.3%), and ureters (30/80; 37.5%). There was no significant association between hemoglobin drop and the number of organs involved (*p* = 0.1734). However, a significant difference was found between the duration of surgery and the number of operated organs (*p* = 0.0176). Specifically, surgeries involving only one organ were significantly shorter than those involving two, three, or four organs (*p* < 0.05). A detailed summary of lesions coexisting with bladder involvement is presented in Table 5.

The proportions of full- and partial-thickness bladder wall infiltration did not differ significantly across groups defined by specific organ involvement (*p* = 0.0768). Similarly, no significant difference was found in the distribution of infiltration depth among groups categorized by the number of organs involved (*p* = 0.6554).

The location of infiltration within the parts of the bladder was not associated with multiorgan involvement of endometriosis lesions (*p* = 0.9337) as shown in Table 6.

### 3.9. Pre-Surgery Complaints

Our next question was what symptoms the patients reported. Specifically, we explored whether pain intensity reflected the size, location, or depth of bladder infiltration, or whether it was unrelated to these features. Was pain more severe in cases with multiorgan involvement or the degree of wall infiltration? A limitation of the study is that most patients, in addition to bladder involvement, also had lesions affecting the large intestine or an adenomyotic uterus. As a result, the overall perception of pain could not be attributed solely to bladder infiltration.

Before surgery, patients reported a range of symptoms. Dysmenorrhea was the most common, affecting 88.7% of patients (VAS range: 1–9), followed by dyschezia in 75.0% (VAS range: 2–9), dyspareunia in 55.7% (VAS range: 2–9), and dysuria in 55.7% (VAS range: 1–9). Additional urinary symptoms included pollakiuria in 17.9%, urinary urgency in 9.0%, hematuria in 2.9%, and urinary incontinence in 3.0%.

The uterosacral ligaments (USL) were infiltrated in 58.8% of bladder endometriosis cases. Patients in this group most commonly reported dyschezia, dysmenorrhea, dyspareunia, and dysuria. In contrast, hematuria, pollakiuria, urinary incontinence, and urinary urgency were less frequently observed. Among symptoms evaluated using the VAS, comparison of scores between patients with USL infiltration and those without USL infiltration revealed a significant difference only for dyspareunia (4.02 ± 3.02 vs. 1.40 ± 2.48; *p* = 0.0021). Differences for other symptoms were not statistically significant: dyschezia (5.22 ± 3.11 vs. 3.65 ± 3.62; *p* = 0.1196), dysmenorrhea (6.65 ± 3.09 vs. 7.00 ± 2.75; *p* = 0.6539), and dysuria (2.65 ± 3.16 vs. 2.65 ± 3.17; *p* = 0.8805).

In patients with additional uterine involvement, dysmenorrhea tended to be more severe, with a near-significant difference in VAS scores compared to those without uterine involvement (7.26 ± 2.81 vs. 6.41 ± 3.07; *p* = 0.0631). However, no significant differences were observed for dyschezia (4.78 ± 3.36 vs. 4.72 ± 3.35; *p* = 0.9473), dyspareunia (2.67 ± 3.05 vs. 3.62 ± 3.11; *p* = 0.2415), or dysuria (3.37 ± 3.15 vs. 2.15 ± 3.07; *p* = 0.1000).

**Figure 3:**
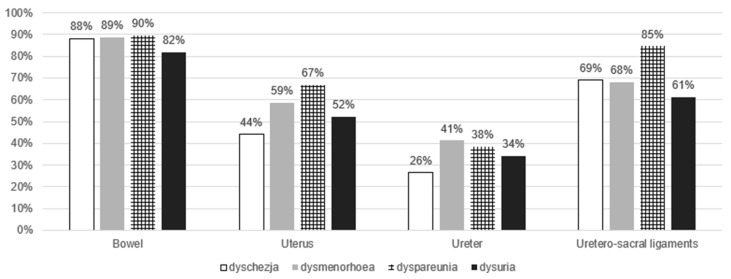
Symptom distribution by organ involvement, regardless of additional lesion sites.

Table 7 summarizes preoperative complications associated with lesions involving the bladder and other organs, while Figure 3 presents the frequency and types of symptoms in patients according to organ involvement, regardless of infiltration at other sites.

### 3.10. Post-Surgery Course

All patients were successfully treated and discharged from the hospital after an average stay of approximately 6 days. Drainage was performed for an average of 3 days, and catheterization lasted approximately 10 days. CRP and PCT levels were measured at three time points, showing an increase from the first to the second measurement, followed by a decrease at the third. Detailed data on hospitalization, drainage, and catheter duration are summarized in Table 8.

CRP levels exhibited significant fluctuations over the three postoperative days (*p* < 0.0001). The lowest concentrations were recorded on Day 1 (44.35 ± 33.77 mg/L), followed by a significant increase on Day 2 (88.21 ± 74.59 mg/L; *p* < 0.05), and a subsequent significant decrease on Day 3 (64.88 ± 61.81 mg/L; *p* < 0.05).

PCT levels showed less variation over time (*p* = 0.0213). The mean value on Day 1 was 0.59 ± 1.67 ng/mL, which slightly increased to 0.66 ± 2.10 ng/mL on Day 2 (not statistically significant). A significant decrease was observed on Day 3 (0.41 ± 1.30 ng/mL) compared to both Day 1 and Day 2 (*p* < 0.05).

Catheterization was significantly shorter for surgeries involving the fundus than for those involving the trigonum (8.07 ± 5.24 days vs. 13.38 ± 1.77 days; *p* < 0.05). Similarly, it was significantly shorter when shaving was compared with resection (8.31 ± 5.18 days vs. 11.69 ± 4.22 days; *p* < 0.05) and when shaving was compared with skinning (8.31 ± 5.18 days vs. 13.00 ± 2.37 days; *p* < 0.05). Catheterization duration decreased with the extent of ureter involvement: it was longest with involvement of two ureters (14.25 ± 0.50 days), followed by one ureter (10.65 ± 4.93 days), and shortest with no ureter involvement (9.90 ± 4.87 days). However, the difference was not statistically significant (*p* = 0.0879).

Drainage lasted the longest in patients with two ureters involved (4.40 ± 3.36 days) and the shortest in those without ureter lesions (2.87 ± 0.94 days). Data on the duration of drainage and catheterization are depicted in Table 9.

### 3.11. Post-Surgery Complications in Bladder Involvement

Postoperative complications were systematically evaluated and reported on a per-patient basis. To ensure clarity in reporting, they were categorized into two main groups: mechanical and functional. Mechanical complications were defined as structural injuries to the urinary tract requiring surgical intervention or prolonged catheterization (e.g., vesicovaginal fistula, ureteral leakage/stenosis). Functional complications were defined as temporary voiding dysfunction, such as urinary retention requiring self-catheterization or prolonged bladder atony, without structural injury.

In total, 9 postoperative complications were observed in 8 patients (10%). Mechanical complications were observed in 6/80 patients, and additionally, functional temporary complications in 3/80 patients. Specifically, early postoperative mechanical complications occurred in 2/80 patients, while late postoperative complications occurred in 4/80 patients.

Mechanical complications were significantly more likely to develop for the full-thickness infiltrations, affect the lower parts of the bladder (particularly the trigonum), and after surgeries where more organs were opened in contrast to functional complications, which did not show such associations. No associations were observed between hemoglobin drop and duration of surgery. When considering all complicated cases, the associations with previous surgery and the number of opened organs were observed. Among 30 patients with prior surgeries, complications occurred in 4 cases (13.3%), whereas among 50 patients without prior surgeries, complications were reported in 2 cases (4.0%). Two complications were observed among patients without any prior surgeries; however, these were the full-thickness lesions, treated by resection, with 3–4 organs involved, and one extended from the bladder trigonum to the posterior wall, and the other, located in the fundus. One complication occurred in a patient with one previous surgery, and three complications were reported in patients with two prior surgeries. No complications were recorded in patients with three previous surgeries.

Details are presented in Table 10.

The complications observed included vesicovaginal fistula in 3 cases, ureterovaginal fistula in 2 cases due to involvement of bladder, uterus, and ureter, and isolated uretero-peritoneal leakage in 1 case. One ureteral stricture was not considered a complication of bladder DE due to the concomitant infiltration of the ureter, which was considered the primary site of DE in this case due to involvement of the bladder and ureter. Vesico–rectal fistulas, recto-vaginal fistula, uretero-rectal fistulas, and vesico–peritoneal leakages were not reported. Functional temporary dysfunction developed in three patients, namely small bladder syndrome in one patient after extensive resection of the bladder, and two temporary hypotony of the bladder, one after trigonum nodule resection, and second after resection of a low bowel nodule and partial resection of USL. The highest complication rate was observed in patients with lesions located in the trigonum (4/6 cases). In all of these patients, two or three additional organs were also involved.

Repair surgery was required in four patients, while in two cases with a vesicovaginal fistula, the complications resolved spontaneously. Surgical techniques included Boari flap reconstruction in one patient after ureter involvement in DE, as well, laparoscopic fistula closure in one patient, secondary ureteral reimplantation into an intestinal segment in one patient (orthotopic bladder). All surgical interventions resulted in complete resolution of complications, yielding a 100% success rate.

Of the six mechanical complications, four occurred in overweight patients and in two patients with normal body weight, suggesting that excess weight may contribute to a more complicated postoperative course. The average BMI among patients who developed complications was 25.38 ± 3.89. Additionally, all of them underwent bowel resection. In five patients, a lesion infiltrated the entire bladder wall, while one had partial infiltration. Higher surgical complexity was observed in all complicated cases. Five patients presented with involvement of three organs (bladder, uterus, and ureter), while one patient had lesions affecting four organs (bladder, uterus, intestine, and ureter). These cases were solved in a bi-disciplinary gynecology–urology team approach. Hemoglobin drop resulted in a hemoglobin difference of 2.65 ± 1.09 g/dL, and the duration of surgery was 332.00 ± 185.39 on average.

## 4. Discussion

Our study confirms that bladder endometriosis is very rarely limited to a single organ and, in the majority of cases, presents as a complex condition, frequently associated with deep infiltration of adjacent organs and a broad spectrum of preoperative symptoms. In 96.3% of cases, bladder involvement coexisted with endometriosis at other sites, most commonly the intestines (87.5%), uterus (61.3%), and ureters (37.5%). Full-thickness infiltration of the bladder wall was observed in 36.4% of patients. A history of prior surgical treatment was reported in 71.8% of cases. The most frequent preoperative symptoms were not characteristic of urinary tract symptoms due to multiorgan involvement and included dysmenorrhea (88.7%), dyschezia (75.0%), dyspareunia (55.7%), dysuria (55.7%), pollakiuria (17.9%), and urinary urgency (9.0%). Patients with USL infiltration experienced significantly greater intensity of dyspareunia. All analyzed cases required multiorgan surgery. Inviting a urologist to participate in surgery allowed us to manage complex situations more effectively. As a bi-disciplinary team, we provided continuous care from initial assessment through follow-up, enabling optimal management of complications and the best possible outcomes. Thus, in our cohort, only eight patients (10%) experienced postoperative complications. Those complications were significantly associated with larger lesion size, full-thickness infiltration, involvement of the bladder trigonum, the number of opened organs, and the history of previous surgery.

Several important reports from other centers have addressed the management of bladder or urinary tract endometriosis [10,11,12,13,14]. Most of these studies are retrospective, single-center analyses. Prospective studies are rare and typically involve very small cohorts [15]. Our study includes a retrospective cohort from the first two years and a prospective cohort from the subsequent three years of analysis. Even multicenter studies have included only moderate sample sizes or focused more broadly on urinary tract endometriosis rather than bladder involvement specifically [16,17,18]. Numerous case reports also exist, often highlighting innovative surgical approaches in individual patients [19,20,21,22,23,24]. Taken together, this observation underscores a significant knowledge gap in the management of complex bladder endometriosis, which most often occurs as part of multiorgan DE and requires comprehensive, multidisciplinary treatment. Treatment remains challenging, and the evidence base is limited. Against this background, our study provides valuable insights by describing in detail a well-characterized cohort of women with bladder endometriosis. Notably, it includes one of the largest known samples to date: 80 consecutively treated patients between 2020 and 2025 in the endometriosis referral center.

Regarding cohort characteristics, the age of our patients is consistent with typical demographics for patients with endometriosis, with a mean age of 37 years. This aligns with the findings of Tomasi et al. [25], who reported an age range of 26 to 44 years in their meta-analysis. Given the interval between disease onset and diagnosis based on the literature, which is 7 to 10 years [26,27], it is likely that these tumors had been growing for a prolonged period and that the patients had unknowingly lived with the condition for an extended time. A key observation in our study is that bladder endometriosis coexisted with endometriosis at other sites in 96.3% of cases. Notably, intestinal involvement was present in 87.5%, even a higher rate than uterine (adenomyosis or endometriosis) involvement (61.3%) and ureteral infiltration (37.5%). This highlights the high complexity of bladder endometriosis, often reflecting widespread pelvic disease. In contrast, Chapron et al. [10] reported a cohort in which each patient had only one site of extra-bladder involvement. In their study, the intestines were affected in 32% of cases, the uterus in 27%, and the ureters in only 9.3%. Other studies have acknowledged multiorgan involvement but often lack a detailed description of the affected anatomical sites.

Symptoms play a critical role not only in the diagnostic process but also in substantially affecting patients’ quality of life, often motivating them to seek medical care. In our study, the most frequently reported complaints were dysmenorrhea (88.7%), dyschezia (75.0%), dyspareunia (55.7%), and dysuria (55.7%). In the literature, symptom reporting is inconsistent. For example, Chapron et al. [10] presented only mean VAS scores without specifying the proportion of affected patients (dysmenorrhea: 7.8 ± 2.5; deep dyspareunia: 6.0 ± 2.9; non-cyclic chronic pelvic pain: 2.8 ± 3.4; gastrointestinal symptoms: 3.1 ± 3.7; LUST: 5.9 ± 3.5). Lertvikool et al. [14] reported that all patients experienced dysmenorrhea, while 72% reported dysuria and 22% hematuria. Similarly, Kovoor et al. [16] noted dysmenorrhea with a mean VAS of 7.8 ± 1.9, dyspareunia (5.6 ± 3.2), chronic pelvic pain (3.2 ± 3.3), dysuria (61.9%), urgency (52.3%), and hematuria (14.3%). In our study, the high frequency of dysmenorrhea and dyschezia likely reflects the advanced complexity of cases. Nearly all patients (96.3%) had endometriosis involving at least one additional site beyond the bladder, with intestinal lesions present in 87.5% and uterine, parametrial, or uterosacral ligament involvement in 61.3%. Consequently, symptoms could not be attributed exclusively to bladder endometriosis. Furthermore, not all patients with bladder involvement experienced dysuria. The varying complexity of disease in different studies may explain discrepancies in symptom profiles. Additionally, symptoms are generally reported collectively for the entire cohort in most studies, making it difficult to determine their association with specific sites of endometriotic involvement.

The type of surgery was individualized based on the affected organs, depth of infiltration, and overall surgical complexity. Lesion size in our cohort ranged up to 9 cm, with a mean of 2.07 ± 1.24 cm, which is larger than those reported in the meta-analysis by Tomasi et al. [25], where lesions reached up to 5.5 cm. In our study, isolated bladder endometriosis was identified in only 3.8% of cases, while full-thickness infiltration of the bladder wall was present in 37.5% of patients. The most commonly performed surgical technique was shaving, used in 45% of cases, followed by full-thickness resection in 40%. In general, shaving or skinning techniques were applied to partially infiltrating lesions, while lesions with full-thickness involvement required resection. Piriyev et al. [11] reported using bladder resection in 88% of cases and shaving in only 24%, while Soares et al. [13] found that 21% of their patients had isolated bladder endometriosis, and bladder shaving without opening the mucosa was performed in 50.7% of cases. Overall, the heterogeneity of clinical presentation and frequent coexisting lesions in other pelvic organs impacted surgical planning and limited the ability to perform a direct comparative analysis of bladder-specific surgical techniques, both in our study and in the findings of other researchers. This aspect is important as we saw that all complications were related to full-thickness disease, which demanded partial bladder resection, so the possibility of performing less radical surgery, such as mucosal skinning or shaving, will significantly reduce the risk of complications.

We also analyzed additional surgical parameters. Operative time was the longest in cases involving large lesions, multifocal bladder involvement, deep infiltrating disease, and patients with a history of previous surgeries (Table 4 and Table 5). In contrast, hemoglobin drop was not significantly associated with these factors, likely reflecting the surgical team’s experience and technical proficiency. In the most complex cases, particularly those with large and difficult localization of lesions, a urologist participated in the procedure in 30% of cases. While urologist involvement was associated with longer operative times, it did not correlate with increased hemoglobin drop. In comparison, Piriyev et al. [11] reported urologist participation in only 7% of cases. Fleischer et al. [28] noted that, although their gynecologists are trained in complex endometriosis surgery, their team prefers to perform bladder endometriosis excisions jointly with a reconstructive urologist whenever possible. Similarly, Rocha et al. [12], in their analysis of urinary tract endometriosis, reported urologist involvement from the beginning in 74.5% of surgeries, with urgent intraoperative consultation required in 3.6% of cases. Urologist participation is also acknowledged by other authors, though without providing any details [14,29,30]. As we mentioned above, this highlights another key aspect of collaborative decision making: volume of different kinds of surgery for gynecologists and urologists and diverse experience of gynecologists and urologists, which, through shared exposure to complex cases, benefits both patients and surgeons.

One theory of bladder endometriosis pathogenesis suggests a secondary, iatrogenic origin following uterine surgeries such as cesarean section or hysterectomy. According to this theory, endometrial cells may enter the bladder through surgical injury to the uterine wall [31,32,33]. In the study by Kovoor et al. [16], only 2 of 21 patients had concurrent cervical adenomyoma, and just 1 of 5 parous women had a history of cesarean section. Piriyev et al. [11] reported previous cesarean delivery in seven patients (3%). In our cohort, 34.6% had prior uterine surgery, including 28.2% with a history of cesarean section (Table 2). Thus, bladder endometriosis following uterine surgery accounted for approximately one-third of cases, suggesting limited support for this theory. A recent systematic review by Salmeri et al. [34] analyzed 81 studies, including 117 women with bladder endometriosis (26 post-cesarean and 91 primary cases). They hypothesized that surgical disruption of the vesicouterine fold and dissemination of decidual fragments might cause bladder lesions. However, their analysis showed that the odds of concomitant pelvic endometriosis were about 16 times lower in post-cesarean cases compared to primary cases (OR 0.06; 95% CI, 0.02–0.20). Despite this, the authors concluded that iatrogenic dissemination remains possible and recommended surgical techniques to reduce this potential long-term complication.

The overall complication rate in the present study was 10%. Factors associated with complications included larger lesions, full-thickness bladder wall infiltration, trigonal location, and history of previous surgeries. Notably, trigonal involvement was linked with longer operative time, greater hemoglobin drop, and larger lesion size (Table 4), and was associated with involvement of at least three organs (Table 6), potentially increasing complication risk. Additionally, surgeries involving the trigonum required significantly longer catheterization (Table 9). Among the 6 mechanical complications, 4 occurred in overweight patients, suggesting that insulin resistance and impaired glucose metabolism may negatively affect wound healing. However, considering the limited sample size of the subgroup with elevated BMI, these observations should be interpreted with caution and require validation in larger cohorts. In comparison, Chapron et al. [10] reported a 2.7% major complication rate (vesico–uterine fistula and intravesical hematoma), while Lertvikool et al. [14] and Pontis et al. [15] observed no intra- or postoperative complications in cohorts with isolated bladder endometriosis. Kovoor et al. [16] noted a 14% major complication rate, mainly related to bowel resection. Rocha et al. [12] reported a 14.4% complication rate, including intestinal fistulas, abscesses, bladder dysfunction, and ureteral injuries. Piriyev et al. [11] observed an 11% complication rate after partial bladder resection, including bleeding and ureteral stenosis, with two cases requiring urological reintervention; no complications occurred after shaving procedures. In our study, no shaving and no mucosal skinning were followed by any complications, so only opening the bladder was connected with such a risk. Considering that 96.3% of surgeries in our cohort involved multiple organs, and 87.5% of patients had additional intestinal lesions that often required bowel resections associated with high complication risk, our low complication rate is noteworthy. For reference, a meta-analysis by De Cicco et al. [35] found a 22.2% overall complication rate for bowel resections in DE, while Ruffo et al. [36] reported 8.3% for laparoscopic approaches, with a 3.2% conversion rate to laparotomy. Overall complication rate due to segmental bowel secretion without bladder surgery ranges from 3.3% to 22.2% [37,38,39,40]. Although some researchers report multiorgan involvement, they do not provide a detailed analysis of complications based on the number and type of organs operated on [41]. The favorable outcomes in our cohort likely reflect the extensive use of preventive intraoperative strategies in our center [42,43], including: non-overlapping suture lines, tissue adhesives, pedicled omental flaps for organ separation (despite mixed views on their efficacy), intraoperative fluorescence with indocyanine green for vascular assessment, two-layer bladder wall suturing, and selective use of prophylactic stitches at bowel anastomoses.

Due to the complexity of procedures required to remove endometriosis infiltrating multiple organs, researchers are concerned about disease recurrence and complications that may require reoperation, with incompleteness of the first operation being a risk factor [44,45]. We found in our study that postoperative complications were associated not only with disease complexity but also with a history of previous surgical interventions. It is well known that re-entering the abdominal cavity carries additional or even higher risk of complications, particularly when adhesions must be released and the same anatomical spaces dissected again. Roman et al. [46] indicated that up to 28% of women may require repeat surgery within 10 years after complete excision of endometriosis. Nirgianakis et al. [47] highlight the role of endometriosis subtype severity in recurrence risk. In our study, 90% of patients with an uncomplicated course reported a marked improvement in quality of life at the 3-month follow-up, and they required no further procedures due to the comprehensive nature of the initial surgery. The patients who experienced complications fortunately had them completely resolved, and at the follow-up visit after completing treatment, they also reported a marked improvement in quality of life. We believe that the presence of a highly experienced urologist, working together with a specialized gynecologic endometriosis team and jointly managing a high volume of complex cases, contributes to lower complication rates. This close collaboration allows for more personalized and preventive surgical planning, supported by the team’s extensive shared experience. Therefore, in our opinion, given the low complication rate achieved for such multiorgan surgery, this approach represented an optimal treatment strategy for these patients.

A multidisciplinary approach involving urologists is widely reported in the literature and is employed to improve outcomes in the surgical treatment of bladder endometriosis and prevent complications [12,48,49,50]. This approach is implemented in our institution in complex, higher-risk patients with urinary tract involvement. While existing studies do not provide direct comparative analyses of outcomes between multidisciplinary teams and gynecologist-only procedures, several authors emphasize the necessity of urological expertise in managing complex cases of bladder endometriosis [11,12,14,28,29,30]. In our cohort, all six elective cases with mechanical complications were managed with the participation of a urologist to have combined experience in solving complex cases, beneficial for both surgeons and patients. Each involved multiorgan disease (at least three organs), with concurrent involvement of the uterus and ureters, and large, multifocal lesions. These surgeries were prolonged (mean duration: 4.5 h), but hemoglobin drop remained comparable to that of uncomplicated cases. Precision and perfect blood control are the huge benefits of a minimally invasive approach, in which access was achieved in all of these cases.

## 5. Limitations

This study has several limitations that should be taken into account when interpreting the findings. The single-center design and the fact that all procedures were performed by one surgeon with a dedicated team, while ensuring consistency and yielding one of the largest known datasets of patients with bladder endometriosis, may limit the generalizability of the results. Additionally, partially, at the beginning of the study, the retrospective design, despite including all consecutive patients treated at our institution, resulted in some missing data. Another limitation is the relatively small number of cases with postoperative complications (n = 8; 10%), which restricted the scope of statistical analysis. However, this low complication rate should also be considered a strength of the study, reflecting the therapeutic success and expertise of the treating reference center.

## 6. Conclusions

Laparoscopic management of bladder endometriosis is feasible and effective, even in cases involving large lesions, prior surgeries, and multiorgan involvement. In nearly all patients (90%), surgery served as both optimal and definitive treatment. These patients had an uncomplicated course, reported marked improvement in quality of life, and required no further procedures because the initial surgery was comprehensive. Although the operation was complex and carried a higher inherent risk than single-organ surgery, the overall risk of one multidisciplinary procedure was ultimately lower than the cumulative risk of multiple staged operations, particularly given the overlap of dissected anatomical compartments. Postoperative complications were primarily associated with disease complexity, including larger lesion size, full-thickness bladder wall infiltration, trigonal involvement, and a history of previous surgical interventions. Optimal outcomes are more likely when procedures are performed by a bi-disciplinary team involving both gynecologists and urologists. Given the heterogeneous clinical profiles of patients with bladder endometriosis, treatment should be carried out in specialized centers where individualized surgical strategies can be implemented to improve patient outcomes.

## Figures and Tables

**Table 1:** Patient characteristics and duration of hospitalization in patients with bladder endometriosis.

Patient Characteristics	Value	Min–Max
Age, years, mean ± SD	36.85 ± 6.18	23–51
Duration of hospitalization, days, mean ± SD	5.75 ± 2.79	2–23
Surgery duration, min, mean ± SD	238.10 ± 95.07	50–580
Hemoglobin drop *, g/dL, mean ± SD	2.54 ± 1.10	0.4–6.8
BMI, kg/m^2^, mean ± SD	23.10 ± 3.33	17–30.80
Underweight, n (%); [<18.5]	6 (7.6%)	17–18.4
Normal weight, n (%); [≥18.5 and <25]	50 (63.3%)	19.1–24.8
Overweight, n (%); [≥25 and <30]	21 (26.6%)	25.5–29.7
Obesity, n (%); [≥30]	2 (2.5%)	30.5–30.8

* Hemoglobin drop was calculated as the difference in the hemoglobin level between baseline and the second day post-surgery. BMI, body mass index; SD, standard deviation.

**Table 2:** Previous surgical interventions in patients with bladder endometriosis.

Type of Prior Surgery	Number of Interventions *							
	0	1	2	3	4	5	6	≥7
Any previous surgery	22	18	13	13	7	1	2	2
Surgery involving the intestines, ureter or any reproductive organ	22	17	11	13	8	1	2	1
Surgery involving the bladder or ureters	70	7	1	1				
Surgery involving the uterus ^†^	51	16	10	1				
Cesarean section	57	11	11					

* Numbers do not add up to 80 due to missing data. † Surgery involving the uterus excluded cesarean sections but included procedures related to endometriosis (e.g., diagnostic laparoscopy, ablation, cystectomy for endometriomas, extensive adhesiolysis) and surgery for uterine fibroids.

**Table 3:** Characteristics of endometriosis tumors of the bladder.

Lesion Location in the Bladder	Diameter (cm)	Degree of Wall Infiltration		Surgery		
	Mean, SD Range	Full n = 30	Partial n = 50	Resection n = 36	Shaving n = 32	Skinning n = 12
Detailed division by location						
Anterior wall n = 5	2.40 ± 0.65 1.5–3	3	2	3	2	0
Anterior/posterior/trigonum n = 1	9.00 -	2	0	1	0	0
Fundus n = 14	1.89 ± 0.94 1–3.5	4	10	4	7	3
Posterior n = 52	1.85 ± 0.92 0.5–4	17	35	18	26	8
Posterior/fundus n = 1	4.5 -	1	0	1	0	0
Posterior/trigonum n = 2	3.25 ± 1.06 2.5–4	2	0	2	0	0
Trigonum n = 5	2.20 ± 0.84 1.5–3.5	2	3	3	1	1
Simplified division by location *						
Fundus n = 15	2.07 ± 1.13 1–4.5	5	10	5	7	3
Trigonum n = 8	3.31 ± 2.46 1.5–9	5	3	6	1	1
Wall n = 57	1.90 ± 0.91 0.5–4	20	37	21	28	8

* Wall category includes only lesions located only in the anterior and posterior walls. The trigonum category includes trigonum, anterior/posterior/trigonum and posterior/trigonum, while the fundus category includes fundus and posterior/fundus.

**Table 4:** Details on hemoglobin drop, surgery duration, and lesion size.

	Hemoglobin Drop *, g/dL		Surgery Duration, min		Lesion Size, cm	
Detailed division by location						
Anterior	3.08 ± 1.26		276.00 ± 95.29		2.40 ± 0.65	
Anterior/posterior/trigonum	2.90		580.00		9.00	
Fundus	2.34 ± 1.13		229.29 ± 92.61		1.89 ± 0.94	
Posterior	2.47 ± 1.07		227.31 ± 83.03		1.85 ± 0.92	
Posterior/fundus	5.00		320.00		4.5	
Posterior/trigonum	3.35 ± 2.19		355.00 ± 176.78		3.25 ± 1.06	
Trigonum	2.34 ± 0.30		197.50 ± 28.72		2.20 ± 0.84	
Simplified division by location ^†^						
Fundus	2.52 ± 1.28	*p* = 0.8141	295.00 ± 114.13	*p* = 0.8117	2.07 ± 1.13	*p* = 0.1138
Trigonum	2.71 ± 1.04		370.71 ± 204.46		3.29 ± 2.66	
Wall	2.53 ± 1.09		231.58 ± 84.38		1.90 ± 0.91	
Division by type of surgery						
Resection	2.52 ± 1.12	*p* = 0.7259	257.42 ± 114.72	*p* = 0.1432	2.88 ± 1.41	*p* < 0.0001
Shaving	2.53 ± 1.21		233.89 ± 79.47		1.46 ± 0.70	
Skinning	2.64 ± 0.737		200.83 ± 74.03		1.75 ± 0.75	
Simplified division by type of surgery						
Resection	2.52 ± 1.12	*p* = 0.6888	257.42 ± 114.72	*p* = 0.1888	2.88 ± 1.41	*p* < 0.0001
Shaving/skinning	2.56 ± 1.10		225.62 ± 78.71		1.53 ± 0.72	
Infiltration						
Full	2.52 ± 1.16	*p* = 0.6188	260.33 ± 115.50	*p* = 0.1286	2.92 ± 1.41	*p* < 0.0001
Partial	2.56 ± 1.08		224.49 ± 78.29		1.56 ± 0.76	
Presence of a urologist during surgery						
Present	2.60 ± 1.17	*p* = 0.6708	273.04 ± 117.37	*p* = 0.0012	2.81 ± 1.61	*p* = 0.1272
Absent	2.52 ± 1.08		223.75 ± 81.16		1.75 ± 0.88	
History of previous surgery						
Any previous surgery	2.38 ± 0.94	*p* = 0.5799	261.07 ± 94.76	*p* = 0.0769	2.12 ± 1.66	*p* = 0.8142
No previous surgeries	2.64 ± 1.20		223.19 ± 96.08		2.00 ± 0.96	
Number of previous surgeries						
No previous surgeries	2.64 ± 1.20	*p* = 0.2760	278.89 ± 119.83	*p* = 0.2213	2.00 ± 0.96	*p* = 0.5529
1 previous surgery	2.24 ± 0.94		330.00 ± 103.07		2.14 ± 1.00	
2 previous surgeries	2.87 ± 0.84		338.75 ± 161.02		2.19 ± 2.84	
3 previous surgeries	1.65 ± 0.78		252.50 ± 3.54		1.75 ± 1.06	

* Hemoglobin drop was calculated as the difference in the hemoglobin level between baseline and the second day post-surgery. ^†^ Wall category includes only lesions located in the anterior and posterior walls. The trigonum category includes trigonum, anterior/posterior/trigonum, and posterior/trigonum, while the fundus category includes fundus and posterior/fundus.

**Table 5:** Summary of surgery complexity due to multiorgan involvement.

Organ	Full/Partial-Thickness Infiltration, n	Hemoglobin Drop *, g/dL, Mean ± SD	Surgery Duration, Min, Mean ± SD
Bladder	2/1	2.00 ± 0.26	116.67 ± 32.15
Bladder, uterus	0/0	-	-
Bladder, intestine	5/14	2.66 ± 1.38	220.53 ± 92.10
Bladder, intestine, ureter	3/4	1.64 ± 1.09	271.43 ± 133.47
Bladder, intestine, ureter, uterus	5/11	2.90 ± 0.88	265.63 ± 87.63
Bladder, uterus, intestine	9/19	2.66 ± 1.05	238.93 ± 60.33
Bladder, ureter	2/0	2.00 ± 0.85	125.00 ± 106.07
Bladder, uterus, ureter	4/1	2.20 ± 0.51	295.00 ± 191.57
One organ	2/1	2.00 ± 0.26	116.67 ± 32.15
Any 2 organs	7/14	2.60 ± 1.34	211.43 ± 94.99
Any 3 organs	16/24	2.41 ± 1.06	250.51 ± 93.19
Any 4 organs	5/11	2.90 ± 0.88	265.63 ± 87.63

* Hemoglobin drop was calculated as the difference in the hemoglobin level between baseline and the second day post-surgery.

**Table 6:** Summary of lesion location in relation to multiorgan involvement.

	Number of Organs			
Location	One Organ	Any 2 Organs	Any 3 Organs	Any 4 Organs
Anterior; n = 5	0	1	4	0
Anterior/posterior/trigonum; n = 1	0	0	1	0
Fundus; n = 14	1	5	5	3
Posterior; n = 52	2	15	25	10
Posterior/fundus; n = 1	0	0	1	0
Posterior/trigonum; n = 2	0	0	1	1
Trigonum; n = 5	0	0	3	2

**Table 7:** Pre-surgery complaints vs. the location of the lesion in the bladder involvement of patients.

Lesion Location	Dyschezia	Dysmenorrhea	Dyspareunia	Dysuria	Hematuria	Pollakiuria	Urinary Incontinence	Urinary Urgency
All patients	51/68 75.0%	63/71 88.7%	39/70 55.7%	44/79 55.7%	2/68 2.9%	12/67 17.9%	2/67 3.0%	6/67 9.0%
VAS	4.74 ± 3.29	6.59 ± 3.02	3.19 ± 3.11	3.06 ± 3.10	-	-	-	-
Bladder alone	1/1 100.0%	1/1 100.0%	1/1 100.0%	3/3 100.0%	1/1 100.0%	1/1 100.0%	1/1 100.0%	0/1 0.0%
VAS	8	9	7	6.00 ± 1.00				
Bladder, intestine	16/18 88.8%	17/18 94.4	10/18 55.6	13/19 68.4	0/17 0.0%	3/17 17.6	0/17 0.0%	1/17 5.9
VAS	5.67 ± 2.93	7.17 ± 2.68	3.00 ± 3.09	3.53 ± 3.06				
Bladder, intestine, ureter	4/7 57.1%	6/7 85.7%	2/7 28.6%	3/7 42.9%	0/7 0.0%	0/7 0.0%	0/7 0.0%	0/7 0.0%
VAS	3.14 ± 3.18	6.57 ± 3.87	1.58 ± 2.70	2.57 ± 3.36				
Bladder, intestine, ureter, uterus	10/12 83.3%	14/14 100.0%	10/13 76.9%	7/16 43.8%	0/12 0.0%	1/12 8.3	0/12 0.0%	0/12 0.0%
VAS	5.92 ± 3.32	8.21 ± 0.98	5.23 ± 3.22	2.50 ± 3.06				
Bladder, intestine, uterus	16/23 69.6%	19/24 79.2%	13/23 56.5%	13/27 48.1%	0/23 0.0%	5/23 21.7%	1/23 4.3%	3/23 13.0%
VAS	4.44 ± 3.26	5.13 ± 3.33	2.70 ± 2.72	2.70 ± 3.11				
Bladder, ureter	0/2 0.0%	2/2 100.0%	0/2 0.0%	0/2 0.0%	0/2 0.0%	0/2 0.0%	0/2 0.0%	1/1 100.0%
VAS	-	7.50 ± 2.12	-	5.00 ± 2.83				
Bladder, uterus, ureter	4/5 80.0%	4/5 80.5%	3/5 60.0%	3/5 60.0%	1/4 25.0%	2/5 40.0%	0/5 0.0%	1/5 20.2%
VAS	3.40 ± 3.36	6.20 ± 3.56	3.60 ± 3.58	3.20 ± 3.96				
USL—all cases	38/47 80.85%	43/49 87.76%	33/47 70.21%	27/51 52.94%	0/46 0.0%	5/46 10.87%	0/46 0.0%	2/46 4.35%
VAS	5.17 ± 3.10	6.59 ± 3.04	4.09 ± 3.02	2.94 ± 3.15				
USL, blader	1/1 100.0%	1/1 100.0%	1/2 50.0%	3/3 100.0%	1/2 50.0%	1/1 100.0%	1/1 100.0%	1/1 100.0%
VAS	8	8	8	7–6				
USL, bladder, intestine	16/18 88.9%	17/18 94.4%	10/18 55.6%	13/19 68.4%	0/17 0.0%	3/17 17.6	0/17 0.0%	1/17 5.9
VAS	5.67 ± 2.9	7.17 ± 2.68	3.00 ± 3.09	3.53 ± 3.06				
USL, bladder, intestine, ureter	4/7 57.1%	6/7 85.7%	2/7 28.6%	3/7 42.9%	0/7 0.0%	0/7 0.0%	0/7 0.0%	0/7 0.0%
VAS	3.14 ± 3.18	6.57 ± 3.87	1.57 ± 2.70	2.57 ± 3.36				
USL, bladder, intestine, ureter, uterus	10/12 83.3%	14/14 100.0%	10/13 83.3	7/16 43.8%	0/12 0.0%	1/12 8.3%	0/12 0.0%	0/12 0.0%
VAS	5.92 ± 3.32	8.21 ± 0.98	5.23 ± 3.22	2.50 ± 3.06				
USL, bladder, intestine, uterus	16/23 69.6%	19/24 79.2%	13/23 56.5	13/27 48.1%	0/23 0.0%	5/23 21.7%	1/23 4.3%	3/23 13.0%
VAS	4.44 ± 3.26	5.13 ± 3.33	2.70 ± 2.72	2.70 ± 3.11				
USL, bladder, ureter	0/2 0.0%	2/2 100.0%	0/2 0.0%	2/2 100.0%	0/2 0.0%	0/2 0.0%	0/2 0.0%	1/2 0.0%
VAS	-	7.50 ± 2.12	-	5.00 ± 2.83				
USL, bladder, ureter, ureter	4/5 80.0%	4/5 80.0%	3/5 60.0%	3/5 60.0%	1/5 20.0%	2/5 40.0%	0/5 0.0%	1/5 20.0%
VAS	3.40 ± 3.36	6.20 ± 3.56	3.60 ± 3.58	3.20 ± 3.96				

VAS, Visual Analog Scale; USL, uterosacral ligament VAS score is calculated as mean ± SD. Numbers are presented as the number of patients reporting a given symptom out of the total number of patients who provided information on that symptom.

**Table 8:** Data on duration of hospitalization, drainage, and catheter and values of inflammatory markers.

Variable	Mean ± SD	Median (Min–Max)
Hospitalization, days	5.75 ± 2.79	5 (2–23)
Drainage, days	3.12 ± 2.32	3 (1–20)
Catheter, days	10.36 ± 4.83	14 (1–15)
CRP 1, mg/L	44.35 ± 33.77	34.9 (7.8–156.10)
CRP 2, mg/L	88.21 ± 74.59	59.55 (5.6–342.00)
CRP 3, mg/L	64.88 ± 61.81	43.20 (3.18–331.62)
PCT 1, ng/mL	0.59 ± 1.67	0.18 (0.03–13.90)
PCT 2, ng/mL	0.66 ± 2.10	0.16 (0.02–14.77)
PCT 3, ng/mL	0.41 ± 1.30	0.12 (0.02–8.57)

CRP, C-reactive protein; PCT, procalcitonin; SD, standard deviation. CRP 1, CRP 2, CRP 3—C-reactive protein concentration on the 1st, 2nd, and 3rd postoperative day. PCT 1, PCT 2, PCT 3—Procalcitonin concentration on the 1st, 2nd, and 3rd postoperative day.

**Table 9:** Details on drainage and catheterization in patients with bladder involvement.

Duration of Drainage and Catheterization	Foley Catheter, Days		Drainage, Days	
Detailed division by location				
Anterior; n = 5	9.80 ± 5.76		2.60 ± 0.55	
Anterior/posterior/trigonum *; n = 1	14		20	
Fundus; n = 14	7.64 ± 5.17		2.86 ± 0.66	
Posterior; n = 52	10.62 ± 4.75		2.98 ± 1.48	
Fundus/posterior; n = 1	14		3	
Posterior/trigonum; n = 2	14.00 ± 0.00		4.00 ± 2.83	
Trigonum; n = 5	13.00 ± 2.24		3.20 ± 1.64	
Simplified division by location ^†^				
Fundus; n = 15	8.07 ± 5.24 ^‡^	*p* = 0.0500	2.87 ± 0.64	*p* = 0.5151
Trigonum; n = 8	13.38 ± 1.77		5.50 ± 6.09	
Wall; n = 57	10.54 ± 4.79		2.95 ± 1.42	
Division by type of surgery				
Resection; n = 32	11.69 ± 4.22	*p* = 0.0038	3.50 ± 3.25	*p* = 0.2316
Shaving; n = 36	8.31 ± 5.18 ^§^		3.14 ± 1.51	
Skinning; n = 12	13.00 ± 2.37		2.50 ± 0.90	
Division by organ				
All patients; n = 80	10.36 ± 4.83	*p* = 0.7796	3.12 ± 2.32	*p* = 0.9022
Bladder alone; n = 3	10.00 ± 6.93		2.67 ± 0.58	
Bladder and ureter; n = 2	8.00 ± 8.49		2.50 ± 0.71	

* The case with anterior/posterior/trigonum localization of endometriosis required the longest duration of catheterization and drainage, due to the extent of the lesion and the type of surgery performed, which involved the augmentation of the bladder. ^†^ Trigonum includes the following localizations of the lesion: trigonum, anterior/posterior/trigonum and posterior/trigonum, while fundus includes fundus and posterior/fundus. ^‡^ Catheterization was significantly shorter between fundus and trigonum (*p* < 0.05). ^§^ Catheterization was significantly shorter between shaving and resection (*p* < 0.05) and between shaving and skinning (*p* < 0.05).

**Table 10:** Comparison of groups with and without complications.

	Any Complications	Mechanical Complications *	Functional Complications
Lesion size, cm, mean ± SD	Yes, 3.79 ± 2.50	Yes, 4.20 ± 2.80	Yes, 3.17 (1.44)
	No, 1.86 ± 0.92	No, 1.91 ± 0.93	No, 2.01 (1.24)
	*p* = 0.0060	*p* = 0.0080	*p* = 0.1077
Hemoglobin drop ^†^, g/dL, mean ± SD	Yes, 2.60 ± 1.30	Yes, 2.65 ± 1.19	Yes, 3.17 (1.92)
	No, 2.54 ± 1.12	No, 2.53 ± 1.11	No, 2.52 (1.09)
	*p* = 0.9701	*p* = 0.9299	*p* = 0.4673
BMI, kg/m^2^, mean ± SD	Yes, 24.03 ± 3.94	Yes, 25.38 ± 4.26	Yes, 23.93 (3.61)
	No, 22.77 ± 3.22	No, 22.84 ± 3.26	No, 22.92 (3.33)
	*p* = 0.4999	*p* = 0.1539	*p* = 0.6217
Duration of surgery, min, mean ± SD	Yes, 322.86 ± 159.03	Yes, 332.00 ± 185.39	Yes, 360.00 (131.15)
	No, 228.94 ± 85.90	No, 231.76 ± 84.44	No, 233.10 (93.11)
	*p* = 0.1790	*p* = 0.3477	*p* = 0.0882
Full-thickness infiltration, %	Yes, 17.2%	Yes, 83.3%	Yes, 3.3%
	No, 6%	No, 33.8%	No, 4.0%
	*p* = 0.1127	*p* = 0.0166	*p* = 0.8800
Location fundus, %	Yes, 10.9%	Yes, 16.6%	Yes, 0.0%
	No, 6.7%	No, 18.9%	No, 4.6%
	*p* = 0.6239	*p* = 0.8925	*p* = 0.3993
Location trigonum, %	Yes, 62.5%	Yes, 83.3%	Yes, 12.5%
	No, 4.2%	No, 4.1%	No, 2.8%
	*p* < 0.0001	*p* < 0.0001	*p* = 0.1724
History of surgery, %	Yes, 20.0%	Yes, 66.7%	Yes, 6.7%
	No, 4.1%	No, 35.1%	No, 2.0%
	*p* = 0.0237	*p* = 0.1273	*p* = 0.2905
Number of affected organs, %	Yes, 3.25 ± 0.46	Yes, 3.17 ± 0.41	Yes, 3.33 ± 0.58
	No, 2.82 ± 0.79	No, 2.84 ± 0.79	No, 2.84 ± 0.78
	*p* = 0.1256	*p* = 0.3026	*p* = 0.2716
Number of operated organs ^‡^, %	Yes, 3.37 ± 0.52	Yes, 3.33 ± 0.52	Yes, 3.67 ± 0.58
	No, 2.86 ± 0.84	No, 2.88 ± 0.84	No, 2.88 ± 0.83
	*p* = 0.0783	*p* = 0.1708	*p* = 0.0900
Number of opened organs ^§^, %	Yes, 3.37 ± 1.19	Yes, 3.50 ± 1.38	Yes, 3.67 ± 2.08
	No, 2.33 ± 1.36	No, 2.35 ± 1.35	No, 2.39 ± 1.34
	*p* = 0.0073	*p* = 0.0211	*p* = 0.1759

* The data in this column are categorized based on the occurrence of complications: the ‘Yes’ group includes cases with complications, while the ‘No’ group includes uncomplicated cases. ^†^ Hemoglobin drop was calculated as the difference in the hemoglobin level between baseline and the second day post-surgery. ^‡^ Number of operated organs was defined as the simple count of distinct organs on which any surgical procedure was performed during the index operation. ^§^ Number of opened organs was defined as the number of organs whose lumen was entered during surgery, either intentionally (e.g., resection) or, less commonly, unintentionally (e.g., perforation).

## Data Availability

Dataset available on request from the authors.

## Abbreviations

The following abbreviations are used in this manuscript:

BMI	body mass index
CRP	C-reactive protein
DE	deep endometriosis
LUTS	lower urinary tract symptoms
MRI	magnetic resonance imaging
PCT	procalcitonin
SD	standard deviation
TVUS	transvaginal ultrasound
USL	uterosacral ligaments
VAS	visual analog scale
